# The Impact of Sex on Changes in Plasma Corticosterone and Cotinine Levels Induced by Nicotine in C57BL/6J Mice

**DOI:** 10.3390/brainsci10100705

**Published:** 2020-10-03

**Authors:** Khoa Nguyen, Keiko Kanamori, Chang Sung Shin, Abdul Hamid, Kabirullah Lutfy

**Affiliations:** 1 Department of Pharmaceutical Sciences, College of Pharmacy, Western University of Health Sciences, 309 East 2nd Street, Pomona, CA 91766, USA; khoa.nguyen@westernu.edu (K.N.); kkanamori.hmri@gmail.com (K.K.); shinc@westernu.edu (C.S.S.); ahamid@westernu.edu (A.H.); 2 Lab Launch, 605 E. Huntington Drive, Suite # 103, Monrovia, CA 91016, USA

**Keywords:** nicotine, cotinine, corticosterone, LCMS, sex, C57BL/6J mouse, CYP2A5, plasma levels, subcutaneous injection

## Abstract

We assessed if there were any sex-related differences in the ability of nicotine to increase plasma corticosterone secretion after single or repeated nicotine administration. For single-dose studies, male and female mice were habituated to the test room for 1 h and injected with saline or nicotine (0.25 or 1 mg/kg, subcutaneously (s.c.)). In repeated-dosing studies, mice were injected with saline or nicotine (1 mg/kg, s.c.) once daily for six days, and, on day 7, received nicotine (1 mg/kg, s.c.). Mice were then euthanized 15 min later, and trunk blood was collected for the measurement of corticosterone, nicotine, and cotinine. Our results showed that saline or nicotine each significantly increased plasma corticosterone levels in both males and females, with a greater response in female mice. Plasma corticosterone levels were increased in male but not female mice after being treated repeatedly compared to single nicotine administration. The level of cotinine, a biomarker of nicotine use, was significantly higher in female than in male mice. Taken together, these novel findings suggest that female mice respond to nicotine and the stress of handling more than male mice and provide for the first-time quantitative data on male–female differences in nicotine-induced elevations of corticosterone and cotinine plasma levels.

## 1. Introduction

Nicotine is the primary component of cigarettes and other tobacco products and it is responsible for the development of addictive behaviors. Some of the addictive properties of nicotine appear to be related to its ability to activate the hypothalamic–pituitary–adrenal (HPA) axis and cause secretion of the stress hormone. When stress is experienced, corticotropic-releasing hormone (CRH) is released from the hypothalamus that then stimulates the release of adrenocorticotropic hormone (ACTH) from the anterior pituitary. ACTH then stimulates the release of cortisol/corticosterone (in humans/rodents) from the adrenal cortex. Our previous study showed that in male C57BL/6J mice, nicotine injections (0.6 mg/kg) elevated plasma ACTH and corticosterone levels 15 min postinjection [1]. Evidence for the involvement of CRH and arginine vasopressin (AVP) in nicotine-mediated elevation of plasma corticosterone was subsequently reported [2]. However, studies are sparse regarding the impact of sex on the nicotine-induced elevation of plasma corticosterone in C57BL/6J mice.

An early study assessed the strain-dependence of the effects of saline and nicotine injections on plasma corticosterone in mice using four different mouse strains, including C57BL/6Ibg mice [3]. These authors reported no consistent sex differences in corticosterone levels following nicotine injections. Subsequent studies, however, showed that basal and stress-induced corticosterone levels are greater in female than in male rodents (reviewed by [4]). Nevertheless, in the widely used C57BL/6J mice, nicotine-induced corticosterone secretion has not been examined in female vs. male mice. Hence, one of the objectives of this study was to measure plasma corticosterone concentrations in adult C57BL/6J male and female mice in response to saline and nicotine administration. We also examined if this response would be altered following repeated nicotine administration and if there would be any sex-related differences in this response.

Nicotine is metabolized to cotinine in the liver by enzymes belonging to the cytochrome P450 family, viz by CYP2A6 in humans [5,6,7,8] and CYP2A5 in mice [8,9,10]. The amino acid sequences of the two enzymes are 84% identical [8], and CYP2A6 and CYP2A5 genes are orthologous. Furthermore, human CYP2A6 and mouse CYP2A5 are very similar in tissue distribution and substrate specificity (reviewed by [11]). Hence, the mouse is an excellent animal model for studying the metabolic pathways and pharmacological effects of nicotine.

The pharmacokinetics of nicotine metabolism in mice depends on mouse strain and gender as well as nicotine dose and its route of administration. While strain-dependence in male mice has been extensively studied [12], data on gender-dependence are sparse. In the widely used C57BL/6 strain, time-course studies of radio-labeled nicotine in the liver after nicotine (1 mg/kg, i.p.) administration [13] showed that females had significantly lower liver nicotine concentrations than males at 5 min post-nicotine administration, suggesting a faster rate of nicotine elimination from the liver of female than male mice. The formation of nicotine-∆5′(1′)-iminium ion, mediated by CYP2A5, is the first step in the formation of cotinine. The in vitro rate of iminium ion formation is significantly faster in female-derived liver microsomes than in their male counterparts [9,14]. These observations strongly suggest that, in vitro, nicotine is metabolized to cotinine at a faster rate in female than in male C57BL mice. However, because females showed high variability in both the in vitro C-oxidation rate and the protein densities of CYP2A5 and its homolog [15], subsequent studies have been mainly limited to male mice, leaving a gap in our knowledge as to whether there are any sex-related differences in this response. For in vivo data too, plasma nicotine and cotinine concentrations resulting from nicotine injection have not, to the best of our knowledge, been investigated in female C57BL/6J mice. Hence, the other aim of this study was to examine the plasma concentrations of nicotine and its stable biomarker, cotinine, in male and female C57BL/6J mice after nicotine administration and to investigate whether there was a correlation between the level of these chemicals and nicotine-stimulated corticosterone secretion.

## 2. Materials and Methods

### 2.1. Subjects

A total of 34 male and 32 female C57BL/6J mice (24–30 g), bred inhouse, were used at the age of 2–4 months. Mice were housed 2–4 mice per cage in disposable plastic cages and maintained on a 12 h light/12 h dark cycle (light on at 6:00 a.m.). All experiments were carried out according to the NIH guidelines for the proper care and use of animals in research and approved by the Institutional Animal Care and Use Committee (R17IACUC013) at the Western University of Health Sciences (Pomona, CA, USA).

### 2.2. Chemicals and Reagents

S(−)-nicotine (1 mg/mL in methanol), (−)-cotinine (1 mg/mL in methanol), (+/−)-nicotine-d_4_ (0.1 mg/mL in acetonitrile), and (+/−)-cotinine-d_3_ (1 mg/mL in methanol) were purchased from Millipore-Sigma (St. Louis, MO, USA). For the in vivo studies, an S(−)-nicotine base was purchased from MP Biomedicals, Inc. (Solon, OH, USA), dissolved in normal saline (sterilized 0.9% sodium chloride in deionized water) and injected subcutaneously (s.c.).

### 2.3. Experimental Design and Procedures

The studies were conducted during the light cycle (between 10:00 a.m. and 2:00 p.m.) in a randomized manner, where animals were randomly assigned to one of the treatments. These studies were not preregistered and were exploratory in nature. The researcher who measured the level of corticosterone and other markers was blind to the treatment until the assay was conducted. No sample calculation was performed. The number of mice used was based on our previous studies [1,2].

### 2.4. Single-Dose Studies

**Scheme 1:**
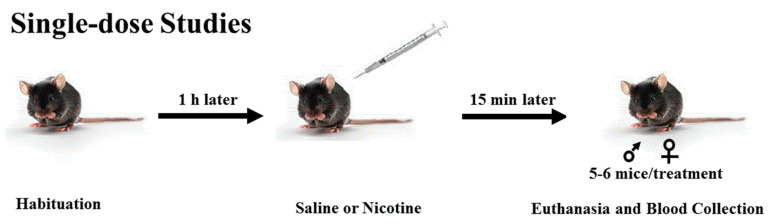
The timeline of single-dose studies.

Mice were brought to the laboratory and allowed to habituate to the test room for 1 h. Mice were then injected with saline or nicotine (0.25 or 1 mg/kg; *n* = 5–6 mice per dose/sex). Fifteen min later, mice were euthanized by cervical decapitation with a pair of sharp scissors, and trunk blood was collected (Scheme 1) in tubes containing 7% ethylenediaminetetraacetic acid. Blood samples were then spun (14,000 rpm) for 10 min, and the supernatant was collected and stored at −80 °C until assayed for plasma corticosterone, nicotine, and cotinine levels, as described below.

### 2.5. Repeated-Dose Studies

**Scheme 2:**
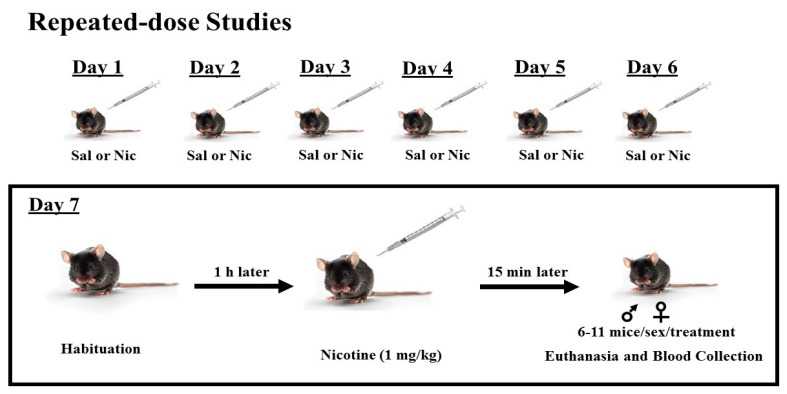
The timeline of repeated-dose studies.

Mice were treated with saline or nicotine (1 mg/kg, s.c.; *n* = 6–11 mice per treatment and sex) once daily for 6 consecutive days. On day 7, mice were brought to the laboratory and allowed to habituate to the test room for 1 h. Mice were then injected with nicotine (1 mg/kg), euthanized 15 min later by cervical decapitation with a pair of sharp scissors, and trunk blood was collected (Scheme 2) in tubes containing 7% EDTA and processed as described above. Plasma samples were then stored at −80 °C until assayed for plasma corticosterone, nicotine, and cotinine levels, as described below.

### 2.6. Corticosterone Measurements

Plasma corticosterone concentrations (5 μL of serum per assay) were measured using a corticosterone enzyme-linked immunosorbent assay (ELISA) kit (Cat. # K014-H5) from Arbor Assays (Ann Arbor, Michigan, USA), according to the manufacturer’s instructions. Optical density at 450 nm was read on a Biotek µQuant microplate reader (Winooski, VT, USA). The standard curve was constructed by the 4-parameter logistic regression fitting routine (online tool from MyAssays), and plasma corticosterone in duplicate was quantified by comparison with the standards.

### 2.7. Nicotine and Cotinine Measurements

Plasma nicotine and cotinine levels were quantified by ultra-performance liquid chromatography–tandem mass spectrometry (UPLC–MS/MS). The analyses were performed on an Acquity UPLC system coupled to a Xevo ToF mass spectrometer (Waters Corporation, Milford, MA, USA), located in the Environmental Analysis Center at California Institute of Technology. The mass spectrometer was operated in positive electrospray ionization mode. MassLynx software (v. 4.1) was used for data acquisition and analyses. Chromatographic separations were performed on an Acquity BEH HILIC column (2.1 × 100 mm, particle size 1.7 μm) protected by a Vanguard precolumn (2.1 × 5 mm). The column temperature was kept at 40 °C. The mobile phase consisted of (A) 10 mM ammonium formate, pH 3.0, and (B) acetonitrile/0.1% formic acid. The analytes were separated with the following gradient program: 0–1.50 min, 5% (A); at 1.55 min, 30% (A); 1.55–2.70 min, 30% (A); 2.80–5.00 min, 60% (A); 5.10 min, return to initial conditions for column equilibration. The flow rate of 0.4 mL/min was kept constant during the 8 min analytical run.

Nicotine and cotinine levels were quantified by multiple reaction monitoring (MRM). The mass spectrometer source parameters and the MRM method were optimized by infusion of neat standards using flow injection analysis. The optimized source/gas parameters were as follows: source temperature, 120 °C; desolvation temperature, 150 °C; desolvation gas flow, 600 L/hr; cone gas flow, 20 L/hr. Table 1 lists the *m*/*z* of the parent ions, the product ions, and the optimized MRM parameters for nicotine, cotinine, and the deuterated internal standards.

### 2.8. Preparation of Standard Solutions

The stock standards were serially diluted with LCMS-grade methanol to prepare working standard solutions. Unlabeled nicotine calibration standards were prepared at 3, 6, 10, 30, 100, 200, and 1000 ng/mL. Cotinine calibration standards were prepared at 3, 10, 30, 100, 300, 600, and 3000 ng/mL. Each standard was prepared as (a) neat standard and (b) as a standard in the plasma matrix where the matrix is plasma from naïve untreated mice. Nicotine or cotinine was first acquired as a single standard to measure the retention time and check sensitivity. Subsequently, nicotine and cotinine were prepared as a mixture of standards (Standards 1 to 7) at the concentrations shown above. The internal standards, prepared at fixed concentrations of 25 ng/mL for nicotine d_4_ and 75 ng/mL for cotinine-d_3_ in acetonitrile, were added to each calibration standard and to the plasma sample, as described below.

### 2.9. Plasma Sample Preparation

The plasma sample was prepared for LC–MS/MS by a validated method [16]. Briefly, 10 µL of each plasma sample was mixed with 80 µL of the internal standards in acetonitrile, vortex-mixed, and centrifuged at 9660× *g* for 25 min at 4 °C to precipitate plasma proteins. This treatment results in a 9-fold dilution of the standard or the analyte and a 1.125-fold dilution of the internal standard. Seventy microliters of the clear supernatant were transferred to a precleaned and dried LCMS vial and saved at 4 °C overnight. The injection volume for LC–MS/MS was 4 µL. To examine whether plasma nicotine and cotinine were preserved during the precipitation of plasma protein by treatment with acetonitrile, recovery of nicotine and cotinine was measured as follows:

Percent Recovery = (Peak area _test_/Peak area _reference_) × (100)

“Test” refers to the addition of a known quantity of standard to the matrix before the addition of acetonitrile and centrifugation, whereas “reference” refers to the addition of the corresponding quantity of standard after acetonitrile treatment and centrifugation of the matrix. In the preparation of these two solutions, we carefully considered the dilution factors described above to achieve the same “nominal” final standard concentration to allow the measurement of recovery.

The quality control (QC) samples containing nicotine, nicotine-d_4_, cotinine, and cotinine-d_3_ at final concentrations (after the dilution described above) of 22.2, 22.2, 66.7, and 66.7 ng/mL, respectively, were acquired every 1.8-h to monitor instrument stability.

### 2.10. Statistical Analyses

Data are expressed as the mean ± standard error of the mean (SEM) of plasma corticosterone, nicotine, or cotinine levels following saline and/or nicotine administration. Data were analyzed using a two-way analysis of variance (ANOVA) or unpaired Student’s *t*-test, whichever appropriate, using GraphPad Prism 8 (GraphPad Software, Inc., San Diego, CA, USA). Fisher’s LSD *post hoc* test was used to reveal significant differences between various groups. A value of *p* ≤ 0.05 was considered statistically significant.

## 3. Results

### 3.1. Corticosterone Secretion was Higher in Female than Male Mice Following Saline or Nicotine Administration

**Figure 1:**
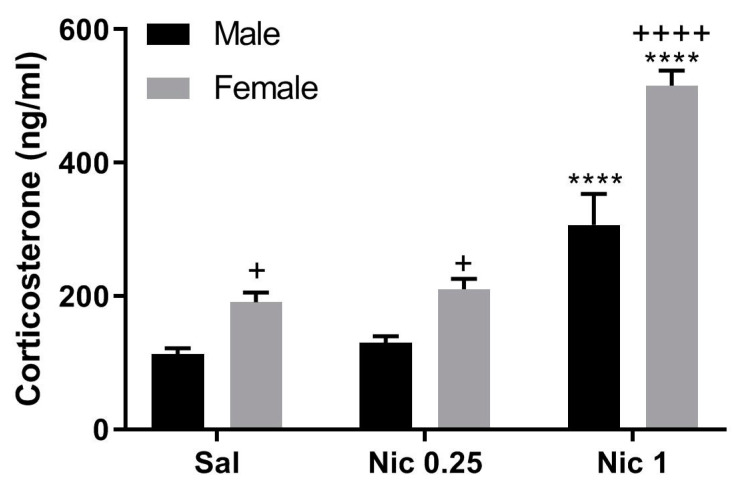
Plasma corticosterone levels following a single saline or nicotine injection in male and female C57BL/6J mice. Mice were habituated to the test room for 1 h, injected with saline or nicotine (0.25 or 1 mg/kg, s.c.), and, 15 min later, euthanized. Blood samples were collected and assayed for plasma corticosterone levels. Data represent mean (± SEM) of corticosterone levels (ng/mL). **** *p* < 0.0001 vs. their respective saline-treated control group; + *p* < 0.05, ++++ *p* < 0.0001 vs. male mice.

Figure 1 shows plasma corticosterone levels following saline or nicotine (0.25 or 1 mg/kg) administration in male and female mice. Two-way ANOVA revealed a significant effect of treatment (F2, 29 = 89.43; *p* < 0.0001), a significant effect of sex (F1, 29 = 49.36; *p* < 0.0001), and a significant interaction between the two factors (F2, 29 = 6.05; *p* < 0.01). The *post hoc* analysis of the data showed that nicotine significantly (*p* < 0.0001) increased secretion of corticosterone in both male and female mice at the high (1 mg/kg) dose but not the low (0.25 mg/kg) dose compared to saline treatment (Figure 1). The *post hoc* test also revealed that female mice had higher plasma corticosterone levels in mice treated with saline (*p* < 0.05) as well as those injected with the low (*p* < 0.05) or high dose (*p* < 0.0001) of nicotine compared to male mice (Figure 1). Together, these results suggest that nicotine compared to saline increased plasma corticosterone levels in both male and female mice. While female mice had higher levels of plasma corticosterone levels than male mice in response to the nicotine challenge, the same difference was observed in saline-treated control mice, suggesting that the sex-related difference observed may not be due only to the nicotine challenge but, rather, female mice are more sensitive to the effect of handling and injection than male mice, at least after a single injection.

### 3.2. Corticosterone Secretion was Higher in Male but not Female Mice after Repeated Nicotine Administration

**Figure 2:**
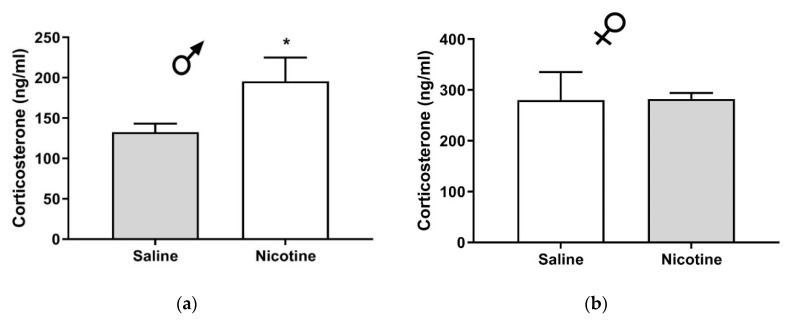
Plasma corticosterone levels following a single nicotine injection in male (**a**) and female (**b**) C57BL/6J mice with prior saline or nicotine exposure. Mice were treated with saline or nicotine (1 mg/kg, s.c.) once daily for 6 consecutive days. On day 7, mice were habituated to the test room for 1 h, injected with nicotine (1 mg/kg, s.c.), and, 15 min later, euthanized. Blood samples were collected and assayed for plasma corticosterone levels. Data represent mean (± SEM) of corticosterone levels (ng/mL). * *p* < 0.05 vs. saline in male mice.

Figure 2 illustrates plasma corticosterone levels following a single nicotine challenge in male (left panel) and female (right panel) mice pretreated with saline or nicotine once daily for 6 consecutive days. Unpaired Student’s *t*-tests of the data in male mice showed a significant (*p* < 0.05) increase in the level of corticosterone following a challenge dose of nicotine in mice with prior nicotine exposure as compared to their saline-treated controls (t = 2.46; df = 15). In contrast, the increase was not observed in female mice pretreated with nicotine compared to their saline-pretreated controls (t = 0.03; df = 12). However, as observed in our single-dose studies, the level of corticosterone was higher in control female mice than male mice (*p* < 0.05). On the other hand, this difference was reduced in female vs. male mice with prior nicotine treatment (*p* > 0.05). Together, these results suggest that prior nicotine treatment enhanced the ability of nicotine to cause the secretion of corticosterone in male but not female mice.

### 3.3. Elevation of Plasma Nicotine and Cotinine after Single Nicotine Injection Assayed by LC–MS/MS

**Figure 3:**
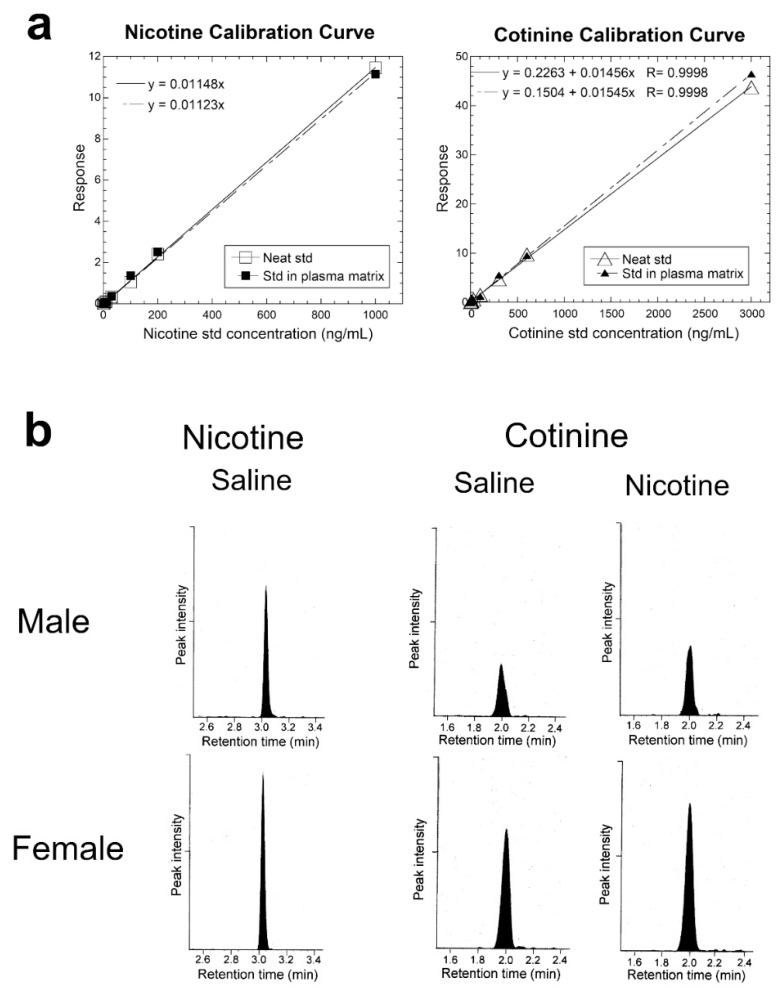
Calibration graphs of nicotine and cotinine by UPLC–MS/MS, shown for the neat standard and standard in plasma matrix (**a**). The linear calibration curve was constructed by plotting response vs. nominal standard concentration, where response = standard peak area * IS conc./IS area and IS are the internal standard of nicotine-d_4_ and cotinine-d_3_, respectively, added at a fixed concentration to monitor instrument stability. The equations of nonweighted least-squares linear regression for the neat standard are very close to those of the standard in the plasma matrix in the concentration range (200–600 ng/mL) where the analyte concentrations were found. Hence, the equations for the neat nicotine and cotinine standards were used to convert the observed response of the respective analyte in the plasma to its concentration. Representative MRM chromatograms of the product ions of plasma nicotine (*m*/*z* 130.06) and cotinine (*m*/*z* 98.06) when the peak area is plotted on the same intensity scale (3400 for nicotine and 1000 for cotinine) for each male/female pair under the same treatment (**b**). Plasma was collected 15 min after nicotine injection (1 mg/kg s.c.) on day 7 following either repeated daily saline (saline) or repeated daily nicotine (nicotine) for 6 days. Chromatograms for nicotine after repeated nicotine treatment are not shown because there was no significant gender difference in the mean nicotine concentrations (as shown in Figure 5a).

Figure 3a illustrates the calibration graphs of nicotine (left panel) and cotinine (right panel) shown as response (standard area * IS conc./IS area) versus concentration, where IS is the internal standard. Good linearity was observed for both the neat standard and for the standard in the plasma matrix, and the equations of least-squares linear regression were very similar (see the figure caption for details). The recovery of nicotine and cotinine after treatment with acetonitrile, examined as described in Materials and Methods, was 102 ± 3.4% for nicotine and 96.3 ± 4.7% for cotinine (mean ± SEM; *n* = 4). Cotinine, with a retention time of 1.98 min, was well-separated from nicotine, with the retention time of 3.02 min. The response of the quality control sample, measured every 1.8 h during the acquisition of the analytes, showed mean ± SEM of 2.06 ± 0.027 (*n* = 6), corresponding to 1.3% variation over time for nicotine, and 8.78 ± 0.2 (*n* = 6), corresponding to 2.2% variation for cotinine. The result demonstrates that the addition of the internal standards ensures reliable quantification of the analytes. Figure 3b shows representative chromatograms of the product ions of plasma nicotine and cotinine when the peak area is plotted on the same peak intensity scale for each male–female pair under the same treatment. The chromatograms illustrate their concentration differences under repeated nicotine injections, which are described below.

**Figure 4:**
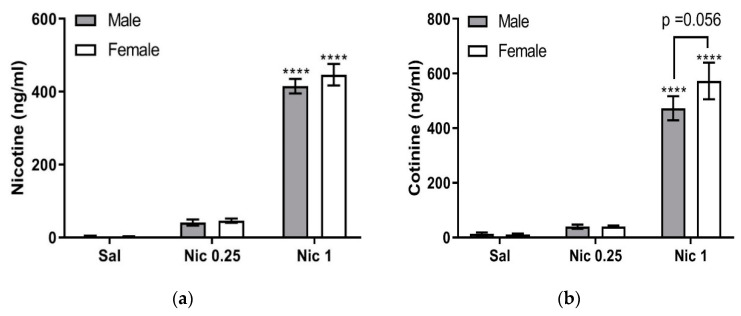
Plasma concentrations of nicotine (**a**) and cotinine (**b**) at 15 min after s.c. injection of a high (1 mg/kg) or low (0.25 mg/kg) nicotine dose in male and female mice. The values are mean ± SEM (*n* = 6, high-dose; *n* = 4, low-dose; *n* = 6, control). **** *p* < 0.0001 vs. their respective control group.

The plasma nicotine and cotinine concentrations of nicotine-treated mice were quantified by comparison of the response of the respective product ion with the standard calibration graph. Figure 4 shows the plasma nicotine and cotinine levels following a single injection of a low (0.25 mg/kg) or high (1 mg/kg) nicotine dose in male and female mice compared to saline-treated controls. For control, given that there was no difference in plasma nicotine and cotinine levels between mice treated with saline or naïve mice, plasma samples of untreated (*n* = 2) and saline-injected (*n* = 4) mice were combined to achieve a total of *n* = 6 for each gender. The level of plasma nicotine (Figure 4a) was elevated to 415.3 ± 19.9 ng/mL in male mice and to 446.8 ± 29.6 ng/mL in female mice after a high-dose nicotine (1 mg/kg) injection (*n* = 6 for each gender). After the low-dose nicotine injection (*n* = 4 per gender), plasma nicotine levels were 40.9 ± 8.3 ng/mL in male mice and 45.8 ± 5.7 ng/mL in female mice. Two-way ANOVA revealed a significant effect of treatment (F2, 26 = 422.1; *p* < 0.0001), but no significant effect of sex (F1, 26 = 0.72; *p* > 0.05) and no interaction between the two factors (F2, 26 = 0.57; *p* > 0.05). The *post hoc* analysis of the data showed that high-dose nicotine (1 mg/kg) significantly increased the plasma nicotine level compared to low-dose nicotine or control in both male and female mice (*p* < 0.0001).

Plasma cotinine (Figure 4b) levels were increased to 472.8 ± 44.0 ng/mL in male mice and to 572.7 ± 66.9 ng/mL in female mice after the high-dose nicotine injection. After the low-dose nicotine injection, plasma cotinine levels were 39.4 ± 7.5 ng/mL in male mice and 40.0 ± 3.4 ng/mL in female mice. Two-way ANOVA revealed a significant effect of treatment (F2, 26 = 125.6; *p* < 0.0001), but no significant effect of sex (F1, 26 = 1.12; *p* > 0.05) and no significant interaction between the two factors (F2, 26 = 1.27; *p* > 0.05). The *post hoc* analysis of the data revealed that high-dose nicotine (1 mg/kg) significantly increased plasma cotinine levels compared to low-dose nicotine or control in both male and female mice (*p* < 0.0001). Both nicotine and cotinine levels were higher in the females than in the males after the high-dose nicotine injection, although this gender difference did not reach statistical significance.

### 3.4. The Level of Nicotine and Cotinine was Higher Following a Challenge Dose of Nicotine in Saline-Pretreated Control Female Mice than Male Mice

**Figure 5:**
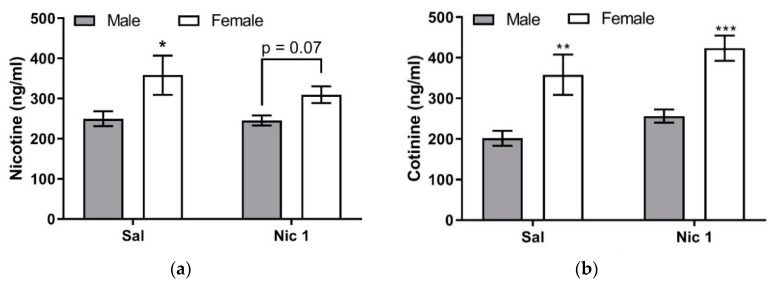
Plasma concentrations of nicotine (**a**) and cotinine (**b**) measured 15 min after a challenge dose of nicotine (1 mg/kg s.c.) given on day 7 in mice pretreated with saline (Saline) or nicotine (1 mg/kg s.c.; Nicotine) once daily for 6 consecutive days. The values are mean ± SEM (*n* = 4–6 mice per sex). * *p* < 0.05 vs. male for nicotine levels; ** *p* < 0.01, *** *p* < 0.001 vs. male for cotinine levels.

Figure 5 illustrates plasma nicotine (Figure 5a) and cotinine (Figure 5b) levels following a nicotine challenge (1 mg/kg) in mice with prior saline or nicotine treatment (once daily for six consecutive days). Two-way ANOVA of plasma nicotine levels revealed a significant effect of sex (F1, 16 = 11.89; *p* < 0.003), but no effect of treatment (F1, 16 = 1.12; *p* > 0.05) and no interaction between the two factors (F1, 16 = 0.78; *p* > 0.05). The *post hoc* test showed that the level of nicotine was significantly higher in saline-pretreated control female mice compared to their respective male mice (*p* < 0.05). Although the level of nicotine was similar in male mice pretreated with saline vs. nicotine (249.9 ± 18.6 versus 245.7 ± 12.6 ng/mL), the plasma nicotine concentrations appeared to be lower, although not significantly different, in female mice with prior nicotine vs. saline exposure (309.9 ± 20.8 ng/mL vs. 358.5 ± 49.0 ng/mL). Because of this reduction, the difference between male and female mice was no longer significant in mice of the two sexes with prior nicotine treatment, but, still, a trend was evident (*p* = 0.07).

Plasma cotinine concentrations were higher in female mice regardless of the pretreatment (Figure 5b). Two-way ANOVA showed a significant effect of sex (F1, 16 = 32.16; *p* < 0.0001) and a significant effect of treatment (F1, 16 = 4.45; *p* = 0.05), but no significant interaction between the two factors (F1, 16 = 0.03; *p* > 0.05). The *post hoc* analyses of the data revealed that, following the challenge dose of nicotine, female mice had higher levels of cotinine compared to male mice regardless of whether they were pretreated with saline (*p* < 0.01) or nicotine (*p* < 0.001), as illustrated by the MRM chromatograms (Figure 3b). Unlike the level of nicotine, the cotinine level appeared to increase in male (201.6 ± 18.7 vs. 256.6 ± 16.2 ng/mL) and female (358.3 ± 49.7 vs. 423.8 ± 31.1 ng/mL) mice with prior nicotine treatment, possibly due to a greater rate of metabolism of nicotine to cotinine over time.

## 4. Discussion

The main novel findings of the present study are that the plasma levels of corticosterone, nicotine, and cotinine were found to be higher in female than male mice. However, the increase in corticosterone level may not be due to changes in plasma nicotine or cotinine levels since the level of corticosterone was also higher in female than male mice treated with saline only. Nevertheless, there appear to be sexual dimorphic changes in the level of corticosterone, nicotine, and cotinine following repeated nicotine treatment. These results suggest that repeated nicotine treatment may differentially regulate the metabolic and/or excretory mechanisms involved in nicotine handling by the body between male and female mice.

The present results show that nicotine-induced elevation of corticosterone is significantly higher in female than male mice. It is noteworthy that, in C57BL/6Ibg mice, females showed significantly higher motor activity, as measured in the Y maze, than males at 10 min after nicotine (1 mg/kg, s.c.) injection [13]. Hence, the observed higher corticosterone level after nicotine challenge in females may be due to both greater motor activity and greater sensitivity to handling. However, our present result clearly shows that nicotine (1 mg/kg) elevated plasma corticosterone to a significantly higher level in control (i.e., mice with prior saline treatment) female mice compared to male mice (Figure 2, left panel), raising the possibility that the sex-related difference observed in the present study cannot be solely attributed to the stress of handling and injection.

The corticosterone concentrations observed in our male mice after single saline or nicotine injections (Figure 1) are reasonably close to the reported values [1,2,17,18,19]. The observed variations in the corticosterone levels reported in these studies are likely to be due to differences in the time of blood collection (10 a.m. or later in our studies and 7–8 a.m. in Pauly’s study) and in the interval between injection and blood collection (15 min in our studies and 25 min in Pauly’s study). Our present work, which extends these studies, shows that in male C57BJ/6J mice, plasma corticosterone was 136.2 ± 18.9 ng/mL upon nicotine challenge with prior saline exposure and 195.6 ± 29.4 mg/mL with prior nicotine exposure (Figure 2). Thus, nicotine significantly increased the level of corticosterone only in male mice with prior nicotine exposure as compared to their saline-treated controls. However, the data need to be interpreted with caution because we did not measure the corticosterone level after saline administration in mice with repeated saline or nicotine treatment, which would have shown if basal corticosterone had been altered in response to repeated nicotine treatment. The lower corticosterone levels after repeated injections (Figure 2) vs. single injection (Figure 1) are likely to be due to habituation to injection-induced stress. Future studies are needed to address this and related issues.

An earlier study [18], using a different saline and nicotine administration protocol, reported that plasma corticosterone levels following an acute nicotine challenge (1 mg/kg) were significantly lower in mice with prior nicotine exposure (2 mg/kg, 3 times/day, for 12 days) than the levels observed in their saline-treated controls. This was attributed to tolerance to the action of nicotine after its repeated administration. In our present work, we observed greater corticosterone secretion in male mice pretreated with nicotine compared to their saline-pretreated controls (Figure 2). A possible explanation is that nicotine injections at a higher dose (2 mg/kg), more frequently (3 times/day), and for a longer duration (12 days) are necessary for nicotine tolerance to emerge, as compared to the enhanced corticosterone level which we observed following the once-daily treatment with a lower dose of nicotine (1 mg/kg) for a shorter period of treatment (6 days) in the present study.

In male C57BL/6J mice, the elimination half-life of plasma nicotine is reported to be 12.9 ± 3.2 min after intraperitoneal nicotine (1 mg/kg) injection [10] and 9.2 ± 1.6 min when the same dose is given subcutaneously [15]. Plasma cotinine has a longer elimination half-life of ~38 min [10,15] and is a more stable biomarker of nicotine use. Plasma nicotine and cotinine are at equilibrium at 15 min after nicotine injection [15], which is the time when our blood samples were collected.

A comparison of the results obtained following single and repeated nicotine treatment suggests that clearance of plasma nicotine in male mice is faster after repeated nicotine injections compared to single injection. The same trend is observed in female mice, where plasma nicotine levels decreased after repeated nicotine injections. The result raises an intriguing possibility that CYP2A5, which catalyzes the metabolism of nicotine to cotinine, undergoes upregulation after repeated nicotine injections. This possibility is in line with our findings that plasma cotinine levels after repeated nicotine injections are higher than the levels found after saline pretreatment in both male and female mice (Figure 5b).

CYP2A5 is induced by a variety of chemicals, and it is generally accepted that a common factor among them is the production of reactive oxygen species, which causes oxidative stress in the liver [20,21]. Recently, Chen and colleagues [22] reported that production of the reactive oxygen species increased by 4-fold upon incubation of microsomes from CYP2A5+/+ mice with nicotine or cotinine, whereas microsomes from CYP2A5−/− mice did not produce any reactive oxygen species. Metabolism of nicotine to cotinine requires two oxidation reactions, namely, the formation of nicotine-∆5′(1′)-iminium ion and its conversion to cotinine.

Cotinine is further oxidized to 3-hydroxycotinine and to cotinine-N-oxide [23] by microsomal CYP2A5. In mice, 3-hydroxy-cotinine is the major elimination product of nicotine (75% of total) followed by cotinine-N-oxide (16%), while nicotine itself represents only 1% in the urine 24 h after nicotine injection [24]. Accordingly, daily nicotine injections are expected to stimulate these oxidation reactions in the liver for disposal not only of nicotine but also of cotinine, with the longer half-life. It is, therefore, possible that repeated nicotine injections for 6 days cause enough oxidative stress in the liver to induce transcriptional upregulation of microsomal CYP2A5. This, in turn, would accelerate the rates of plasma nicotine and cotinine disposal and result in lower plasma nicotine and cotinine in mice that received repeated nicotine injections (Figure 5) compared to those that received nicotine for the first time and, therefore, have constitutive CYP2A5 levels (Figure 4). However, it is not currently known whether oxidative stress in the liver caused by repeated nicotine injections induces transcriptional upregulation of CYP2A5. This possibility, raised by our current study, is an intriguing avenue for future investigation.

The novel aspect of the present work is that significantly higher plasma cotinine concentrations were observed in female mice compared to male C57BL/6J mice after single or repeated nicotine treatment (Figure 5b). The higher plasma cotinine concentration observed in our female mice after one-time nicotine injection (i.e., in mice pretreated with saline; Figure 5b) may be, at least partly, due to the higher plasma nicotine concentrations in female mice compared to male mice (Figure 5a). However, because plasma cotinine is significantly higher in female than male mice under repeated nicotine treatment (Figure 5b), whereas plasma nicotine in female mice is not significantly higher than male mice (Figure 5a), it is more likely that female-specific regulation of CYP2A5 expression also contributes to cotinine formation. To the best of our knowledge, plasma cotinine concentrations after nicotine injections have not been previously measured in female C57BL/6J mice for comparison with male mice.

In male mice, there is a strain-specific genetic variation in CYP2A5 in the liver. For example, two common mouse strains, DBA/2 and C57BL/6, differ at a single nucleotide codon, T and C, respectively, at the second position of the 117 codons in CYP2A5. This results in amino acid substitution of Val (in DBA/2) by Ala (in C57BL/6), which reduces its coumarin 7-hydroxylase activity (often used in early assays of CYP2A5) in C57BL/6 [15]. In nicotine metabolism, the time-courses of nicotine concentration in the plasma after s.c. injection of 1 mg/kg nicotine was similar between the two strains. Upon s.c. injection of cotinine, the half-life of plasma cotinine was considerably longer in DBA/2 than in C57BL/6, an effect that was suggested to be due to differences in the renal disposition of cotinine [15]. Therefore, in view of the strain-specific genetic variations, our present work focused on one widely-used mouse strain, C57BL/6J. Our C57BL/6J mice, as well as most mice referred to in this paper [12,15], are inbred. It is noteworthy that male C57BL/6 [15] and C57BL/6Ibg [12] show similar plasma nicotine pharmacokinetics; after injection of 1 mg/kg nicotine, these mice showed plasma nicotine half life at 9.2 ± 1.6 and 6.85 ± 0.49 min, respectively, and cotinine half life at 23.7 ± 2.0 and 20.1 ± 2.3 min, respectively. Given that these values are not significantly different from each other in the two inbred mouse lines, we believe that the mutation in the CYP2A5 gene that impacts nicotine disposition may be rare in inbred C57BL/6 mice.

For the male–female difference within this strain, analysis of hepatic CYP2A5 protein levels in female mice is difficult due to the presence of a female-specific CYP2A4 protein that is 98% identical to CYP2A5 in the amino acid sequence and indistinguishable by Western blot. However, CYP2A4 has a 15-fold lower affinity for nicotine compared to CYP2A5 and makes little contribution to nicotine metabolism in vivo [8]. These considerations strongly suggest that the sex differences in nicotine effects in the C57BL/6 strain reported in this study are primarily associated with male–female differences in the regulation of hepatic CYP2A5, for example, by the female hormone, estradiol, which is discussed below.

In females, an additional mechanism of regulation of CYP2A5/CYP2A4, viz by female hormones, may occur. It has been suggested that the observed wider ranges of CYP2A5/CYP2A4 protein levels and of the in vitro C-oxidation rates in female C57BL/6 mice compared to male mice [14,15] is due to the effect of female sex hormones, estradiol and progesterone, whose concentrations vary during the estrous cycle of 3–4 days [25,26]. Although we did not examine possible effects of the estrous cycle on the rate of cotinine formation, the current result is less likely to be largely affected by fluctuations in female hormone levels because we collected samples from five mice that received nicotine for seven days, which encompass most if not all phases of the estrous cycle. However, future studies are needed to examine if the increase in cotinine levels is due to hormonal changes during different phases of the estrous cycle.

The possible effect of female hormones on nicotine/cotinine metabolism in humans has been reported [27]. In primary human hepatocyte culture, the expression of CYP2A6, as measured by mRNA level, was enhanced at estradiol and progesterone concentrations found during pregnancy [28]. For mouse CYP2A5, possible regulation by estradiol or progesterone, which affects the nicotine C-oxidation rate, has not been investigated to date. Future studies are needed to clarify whether or not nicotine regulates the expression and activity of CYP2A5 in C57BL/6J mice, and whether estradiol and progesterone at physiological concentrations contribute to the observed sexual dimorphism in nicotine/cotinine changes between male and female C57BL/6J mice.

## 5. Conclusions

The current results provide, for the first time, quantitative data on male–female differences in nicotine-induced corticosterone elevation. Likewise, sex-related changes in the levels of nicotine and cotinine between male and female mice are another novel aspect of the present study. However, the former changes may be due to the stress of handling and injection than changes in plasma nicotine or cotinine levels. While the present study reports differences in the level of nicotine and its metabolite between male and female mice, additional experiments are needed to confirm the sex-related differences observed in the present study. For example, we need to assess the impact of different phases of the estrous cycle on this sex-related difference. Additionally, we need to assess the impact of the removal of the gonads on this sex-related difference. Furthermore, this result needs to be confirmed by studies using receptor antagonists to block the action of estrogen and progesterone.

## Figures and Tables

**Table 1:** Multiple reaction monitoring (MRM) parameters for analytes and their respective internal standards.

	[M+H]^+^ (*m*/*z*)	Product Ion (*m*/*z*)	Cone Voltage (V)	Collision Energy (eV)
Nicotine	163.1	130.06	34	20
Nicotine-d_4_	167.1	134.1	32	20
Cotinine	177.1	98.06	40	20
Cotinine-d_3_	180.1	101.08	44	22
